# Maternal diet of polyunsaturated fatty acid altered the cell proliferation in the dentate gyrus of hippocampus and influenced glutamatergic and serotoninergic systems of neonatal female rats

**DOI:** 10.1186/s12944-016-0236-1

**Published:** 2016-04-05

**Authors:** Mimi Tang, Min Zhang, Hualin Cai, Huande Li, Pei Jiang, Ruili Dang, Yiping Liu, Xin He, Ying Xue, Lingjuan Cao, Yanqin Wu

**Affiliations:** Institute of Clinical Pharmacy & Pharmacology, Second Xiangya Hospital, Central South University, Changsha, Hunan 410011 PR China; School of Pharmaceutical Sciences, Central South University, Changsha, China; Institute of Clinical Pharmacy, Jining First People’s Hospital, Jining Medical University, Jining, 272000 PR China

**Keywords:** Polyunsaturated fatty acids (PUFAs), Arachidonic acid (AA), Docosahexaenoic acid (DHA), Neurogenesis, Neurotransmitter system

## Abstract

**Background:**

Long-chain polyunsaturated fatty acids (PUFAs) are major components of the phospholipids that forming the cell membrane. Insufficient availability of PUFAs during prenatal period decreases accretion of docosahexaenoic acid (DHA) in the developing brain. DHA deficiency is associated with impaired attention and cognition, and would precipitate psychiatric symptoms. However, clinical studies on the potential benefits of dietary DHA supplementation to neural development have yielded conflicting results.

**Methods:**

To further investigate the neurochemical influence of maternal PUFAs levels, we assessed the functioning of various neurotransmitter systems including glutamatergic, dopaminergic, norepinephrinergic and serotoninergic systems in the brain of neonatal female rats by HPLC-MS/MS. Meanwhile, the cell proliferation of neonatal rats was investigated using immunefluorescence.

**Results:**

Different maternal n-3 PUFAs dietary influenced the FA composition, cell proliferation in the dentate gyrus of hippocampus and the contents of γ-aminobutyric acid (GABA), glutamine (GLN), dopamine (DA) and its metabolites [3,4- dihydroxyphenyl acetic acid (DOPAC) and homovanillic acid (HVA)], norepinephrine (NE), vanilmandelic acid (VMA) and 5-HT turnover in the brain of neonatal rats. However, the mRNA expression of key synthase of neurotransmitters remains stable.

**Conclusions:**

Our study showed that maternal deficiency of n-3 PUFAs might play an important role in central nervous system of neonatal female rats mainly through impairing the normal neurogenesis and influencing glutamatergic system and 5-HT turnover.

**Electronic supplementary material:**

The online version of this article (doi:10.1186/s12944-016-0236-1) contains supplementary material, which is available to authorized users.

## Background

Polyunsaturated fatty acids (PUFAs) are major components of the phospholipids that form the cell membranes of tissues and thus play an important role in the structure of neuronal cells in the central nervous system, where they are of the highest concentrations [1]. The lipid requirements of young infants, particularly the requirements for the n–6 and n–3 fatty acids for brain growth and development, are currently an area of intense interest. As indicated in previous studies, accumulation of large amounts of arachidonic acid (20:4n–6, AA) and docosahexaenoic acid (22:6n–3, DHA) in the membranes of the brain during late prenatal and early postnatal is essential for its development [2]. To the contrary, variation in brain FA composition, decreased DHA in specific, affects neurodevelopment, alters visual, attention and cognitive functions, and exhibited symptoms of anxiety, aggression and depression in animals models [3]. Previous findings demonstrated that the link between n-3 fatty acid deficiency and vulnerability to depressive symptoms is more robust in females [4], which suggested that gender differences might be involved in the pathophysiology of n-3 fatty acid deficiency induced neurodevelopment impairment. Therefore, one of our aims in the present study is to determine the effects of maternal n-3 PUFAs deficiency or supplementation on brain FA composition in neonatal female rats.

The mechanisms underlying n-3 PUFAs deficiency induced neurodevelopment impairment are still unclear. One of possible mechanisms may be related to its effects on multiple neurotransmitter systems. N-3 PUFAs has been shown to regulate many of the neurobiological systems, such as glutamatergic system, dopaminergic system, noradrenergic system and serotonergic system [5], and related receptors. Animal studies suggested that reduced brain DHA could induce degradation of glutamatergic transmission in hippocampus of aging rats [6]. As an important part of glutamine(GLN)/glutamate(GLU) (gamma-aminobutyric acid, GABA) cycle (GGC), dishomeostasis of GLN might contribute to the changes in GLUergic or GABAergic system [7]. Additionally, inadequate n-3 PUFAs diet was reported to alter densities of dopamine receptors, including D1 and D2 [8]. Moreover, n-3 PUFAs diet may also have an effect on 5-HT turnover [9]. To further understand how the development of central nervous systems are affected by the availability of dietary n-3 PUFA, the other aim of the current study is to determine the expression of the key enzymes involved in the neurotransmitter metabolism and the levels of different neurotransmitters and their metabolites in the brain of rats. Then, we used Pearson correlation analysis to evaluate possible associations between certain FA and neurotransmitters. Cell proliferation in dentate gyrus of hippocampus was also investigated in the present study.

## Results

### Maternal deficiency of PUFAs decrease the cell proliferation in neonatal female rats

**Fig. 1:**
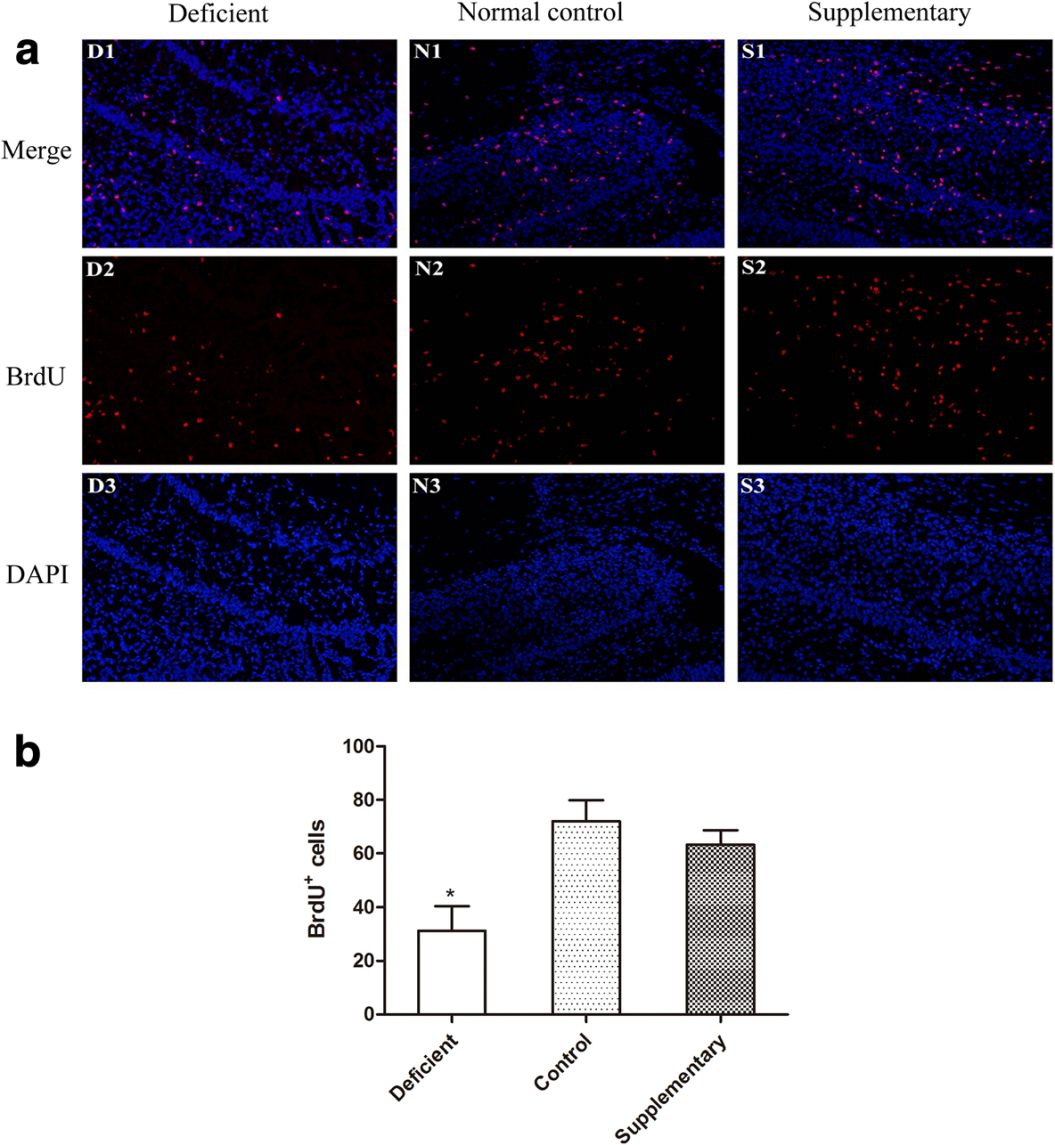
Maternal n-3 PUFAs deficiency decreases the neurogenesis in dentate gyrus (DG) region of neonatal hippocampus. BrdU was examined at the DG region by immunofluorescence (**a**); Quantification of BrdU at the DG region of neonatal hippocampus (one-way ANOVA, TUKEY post hoc test, *p < 0.05 vs. Control) (**b**). *n* = 5 in each group

Newborn cells in the developing rat dentate gyrus can be quantified by immunostaining of BrdU incorporated into the nuclei of dividing cells [10]. The effect of intraperitoneal injection on dentate gyrus neurogenesis was evaluated by comparing the average number of BrdU-positive cells per area in control. ANOVA analyses showed that cell proliferation in neonatal female rats was significant decreased in Deficient group than that in Control group (*P* < 0.01, Fig. 1).

### Fatty acid composition in liver

Treatment of maternal rats with the deficient diet decreased the percentage of C18:0 (stearic acid), C18:1n9c (oleic acid), C22:6n3 (DHA), total n-3 PUFA and the ratio of n-3/n-6 and DHA/AA, and increased the percentage of C16:0 (palmitic acid), C18:2n6c (methyl linoleate), C20:4n6 (AA), C24:0 (tetracosanoic acid) and total n-6 PUFA in the livers of neonatal female rats compared with control group. High (n-3) content in the maternal diet resulted in decreased levels of C16:0, C18:1n9c, C18:2n6c, AA and total n-6 PUFA, and increased levels of C18:3n3, C20:5n3, C22:5n3 (EPA), DHA and total n-3 PUFA compared with Control offspring. The ratio of n-3/n-6 rose about eightfold and the ratio of DHA/AA increased sixfold in the Supplementary group compared to Control (Shown in Table 1).

**Table 1:** Effects of maternal dietary (n-3) PUFA treatment on liver fatty acid composition of 1^st^-litter neonatal female rats

FA	Percent of Total FA (area percent)		
	Deficient	Control	Supplementary
C14:0	1.0 ± 0.1	1.0 ± 0. 0	1.0 ± 0.2
C16:0	27.1 ± 0.5	26.3 ± 1.2	22. 3 ± 1.2*
C16:1	1.4 ± 0.1	1.6 ± 0.0	1.6 ± 0.2
C18:0	6.7 ± 0. 4*	9.5 ± 0.8	8.2 ± 0.6
C18:1n9c	11.6 ± 0.6**	18.5 ± 0.8	12.6 ± 0.7**
C18:2n6c	20.8 ± 1.3	16.6 ± 0.5	12.4 ± 0.9*
C18:3n3	0	0	0.8 ± 0. 1*
C20:4n6	16.8 ± 1.0*	13.5 ± 0.5	6.1 ± 0.4**
C20:5n3	0	0.5 ± 0. 0	4.0 ± 0.5**
C24:0	3.0 ± 0.1**	1.6 ± 0. 2	1.5 ± 0. 2
C22:5n3	0	0	3.605 ± 0. 3*
C22:6n3	0.8 ± 0. 2*	4.0 ± 0.5	11.1 ± 1.1**
Other MUFA	1.7 ± 0. 1**	2.6 ± 0. 1	1.9 ± 0. 1**
Other n-6 PUFA	3.7 ± 0. 4*	1.0 ± 0. 1	0
Total n-3 PUFA	0.7 ± 0. 2**	3.9 ± 0.5	21.0 ± 0.2**
Total n-6 PUFA	40.7 ± 1.6**	30.7 ± 0. 4	18.8 ± 1.4**
n-3 PUFA/n-6 PUFA	1.7 ± 0.6**	12.7 ± 1.6	107.3 ± 7**
DHA/AA	0.04 ± 0.01**	0.31 ± 0.03	1.95 ± 0.08**

Liver FA Composition was expressed as mean ± SEM (*n* = 5-6 per group). Data were analyzed by variance (ANOVA) for multiple comparisons followed by TUKEY or Dunnett’s t test for post-hoc test. ** *p* < 0.01, * *p* < 0.05 vs. Control

### Fatty acid composition in brain

Maternal deficient n-3 diet decreased the percentages of C18:1n9c, DHA, total n-3 PUFAs, and the ratio of n-6/n-3 as well as DHA/AA, and increased the percentage of C18:0, total n-6 PUFAs in the brain of neonatal female rats compared with control group. High (n-3) content in the maternal diet resulted in decreased levels of AA, total n-6 PUFAs and increased levels of C18:1n9c, DHA and total n-3 PUFAs compared with Control offspring (Shown in Table 2). Interestingly, the ratio of n-3/n-6 and DHA/AA only increased two times in the Supplementary group compared to the Control group, which is far less than the proportion in liver.

**Table 2:** Effects of maternal dietary (n-3) PUFA treatment on whole brain fatty acid composition of 1^st^-litter neonatal female rats

FA	Percent of Total FA (area percent)		
	Deficient	Control	Supplementary
C14:0	2.4 ± 0.1	2.1 ± 0.2	2.5 ± 0.0
C16:0	42.5 ± 1.3	39.7 ± 1.1	40.5 ± 0.5
C16:1	3.0 ± 0.0	4.448 ± 0.1	4.5 ± 0.1
C18:0	20.8 ± 0.4*	19.7 ± 0.2	20.2 ± 0.1
C18:1n9c	12.1 ± 0.1**	13.2 ± 0.1	13.7 ± 0.2*
C20:4n6	9.2 ± 0.3	9.4 ± 0.3	7.1 ± 0.5**
C22:6n3	0**	5.3 ± 0.2	7.3 ± 0.3**
Other MUFA	2.9 ± 0.0**	3.1 ± 0.0	3.2 ± 0.1
Other n-6 PUFA	6.2 ± 0.2**	1.5 ± 0.1	0
Total n-3 PUFA	0**	5.2 ± 0.2	7.3 ± 0.4**
Total n-6 PUFA	15.4 ± 0.5**	10.7 ± 0.7	7.2 ± 0.5**
n-3 PUFA/n-6 PUFA	0**	49.2 ± 1.6	105.3 ± 10.2**
DHA/AA	0**	0.56 ± 0.02	1.07 ± 0.07**

Brain FA Composition was expressed as mean ± SEM (*n* = 5-6 per group). Data were analyzed by variance (ANOVA) for multiple comparisons followed by TUKEY or Dunnett’s t test for post-hoc test. ** *p* < 0.01, * *p* < 0.05 vs. Control

### Brain neurochemistry and gene expression of related enzyme

**Fig. 2:**
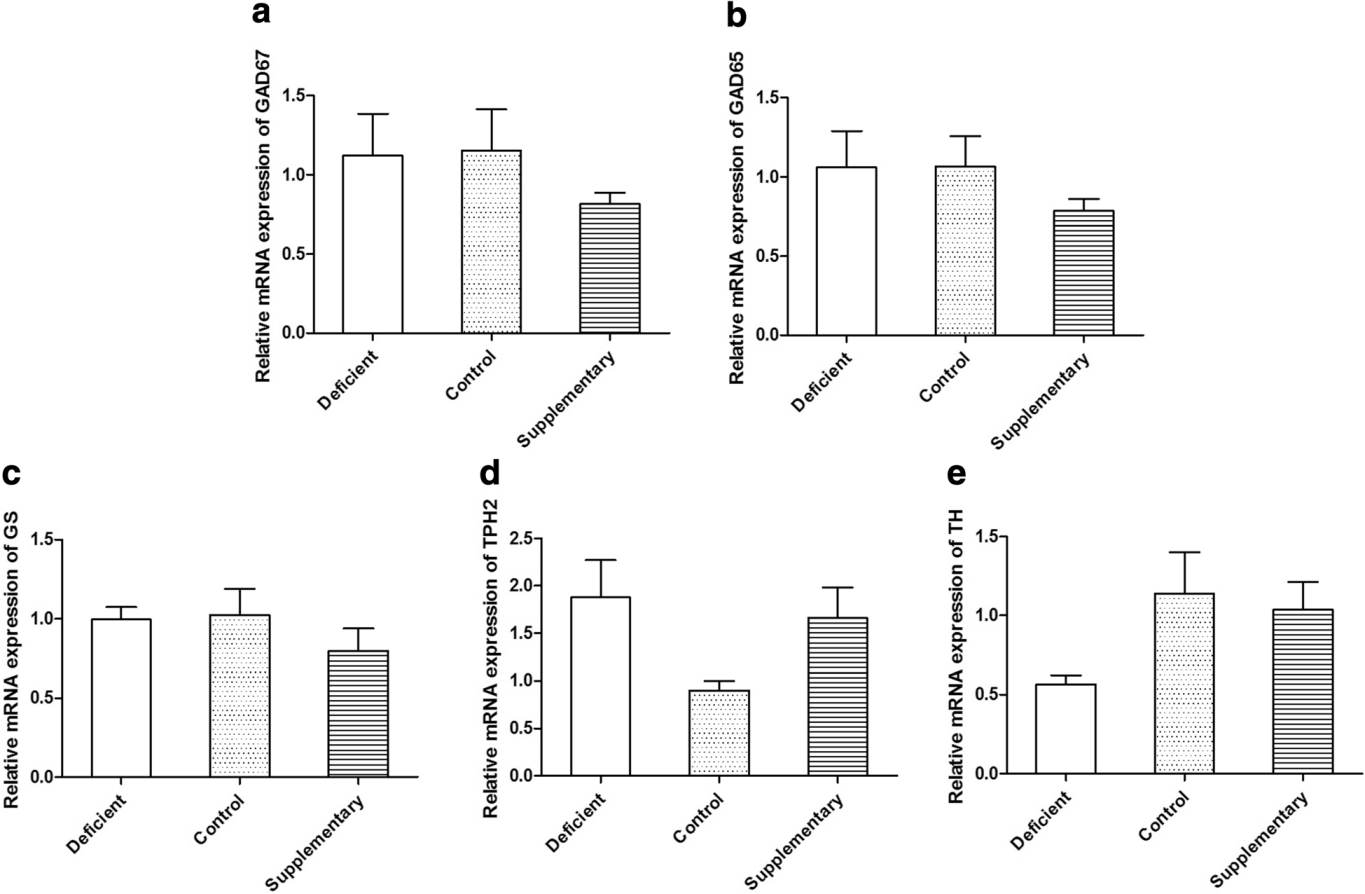
Effect of maternal diet of fatty acid on mRNA levels of the metabolizing enzymes of the neurotransmitters in brain of neonatal rat. Relative mRNA expression of GAD67 (**a**); Relative mRNA expression of GAD65 (**b**); Relative mRNA expression of GS (**c**); Relative mRNA expression of TPH2 (**d**); Relative mRNA expression of TH (**e**)

Maternal n-3 PUFAs deficiency resulted in significant increase of γ-aminobutyric acid (GABA) status (Table 3, *p* < 0.01) and decrease of glutamine (Gln, Table 3, *p* < 0.01) in the brain of neonatal offspring. Correspondingly, maternal n-3 PUFAs supplementation increased the Gln level in the brain of neonatal rats whereas the level of GABA remains unchanged. However both maternal deficient and supplementary in n-3 PUFAs did not alter the level of glutamic acid (Glu) and the expression of glutamate decarboxylase (GAD) 67, GAD65 and glutamine synthetase (GS) (Fig. 2a-c), which are crucial for the biosynthesis of GABA. GABA is produced in the CNS via decarboxylation of GLU, in a reaction catalyzed by glutamic acid decarboxylase (GAD). Within the CNS, the majority of GLU is produced from glutamine (GLN) via the enzyme glutaminase. Regarding the dopamine system, maternal deficient diets did not alter the level of DA (Table 3.) and the expression of the key DA synthase emzyme tyrosine hydroxylase (TH, Fig. 2e) in the brain of neonatal female rats, however the DA metabolites [3,4- dihydroxyphenyl acetic acid (DOPAC) and homovanillic acid (HVA)] increased significantly (Table 3. *p* < 0.01). Female rats whose mother maintained on n-3 PUFAs Supplementary diet exhibited significant deficits in DA levels and surplus in DOPAC relative to Control group, but the mRNA levels of TH remain stable. Supplementary group also exhibited decreased norepinephrine (NE), without altering the metabolites vanilmandelic acid (VMA) and 4-Hydroxy-3-methoxyphenylglycol (MHPG). Both maternal deficient or supplementary diet had no effect on tryptophan (TRP) and serotonin (5-HT) levels and did not affect the expression of tryptophan hydroxylase 2 (TPH2) (Fig. 2d). But Supplementary group exhibited increased kynurenic acid (KYN) compared with Control. Intriguingly, the metabolite of 5-HT, 5-hydroxy indole acetic acid (5-HIAA), and the ratio of 5-HIAA to 5-HT, were generally enhanced in supplementary group, and decreased in deficient group.

**Table 3:** The content of major neurotransmitters and their metabolites in whole brain of 1^st^-litter neonatal female rats

Compound	Deficient	Control	Supplementary
GABA (ug/g)	273.2 ± 7.29**	225.0 ± 7.9	220.7 ± 14.1
GLU (ug/g)	604.8 ± 33.1	580.5 ± 56.2	550.7 ± 50.6
GLN (ug/g)	264.7 ± 18.1**	422.1 ± 32.4	788.5 ± 53.2**
DA (ng/g)	98.2 ± 10.8	106.4 ± 18.5	49.8 ± 4.5**
DOPAC (ng/g)	4848.6 ± 286.9**	3797.4 ± 178.8	4407.3 ± 221.5*
HVA (ng/g)	11092.5 ± 963.5**	7839.8 ± 908.1	9236.3 ± 988.0
NE (ng/g)	2026.9 ± 409.7	2240.3 ± 323.5	1046.8 ± 106.5**
MHPG (ng/g)	2844.8 ± 185.3	2583.5 ± 245.3	2870.5 ± 225.2
VMA(ng/g)	18152.5 ± 1482.2**	24125.9 ± 1439.0	26581.2 ± 1395.4
TRY (ug/g)	31.0 ± 1.9	30.8 ± 3.0	45.1 ± 3.4
5-HT (ng/g)	215.9 ± 32.3	161.8 ± 20.9	169.2 ± 14.3
5-HIAA (ng/g)	174.1 ± 19.2	223.8 ± 25.5	627.8 ± 41.7**
5-HIAA/5-HT	0.618 ± 0.1**	1.745 ± 0.3	4.226 ± 0.6**
KYN (ng/g)	507.2 ± 49.8	487.2 ± 35.6	626.0 ± 26.8**

Data were expressed as mean ± SEM (*n* = 6-8per group). Data were analyzed by variance (ANOVA) for multiple comparisons followed by TUKEY or Dunnett’s t test for post-hoc test. ** *p* < 0.01, * *p* < 0.05 vs. Control

### The correlation of FA composition and neurotransmitter concentration in brain of neonatal rat

**Fig. 3:**
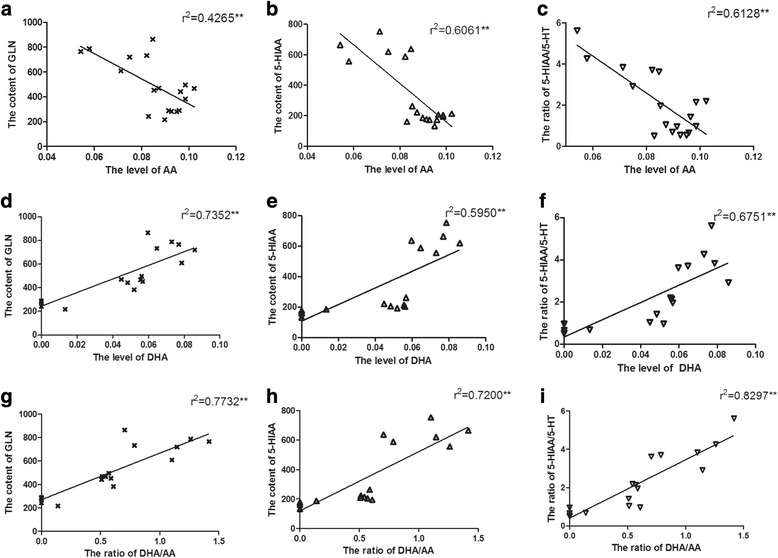
The correlation of FA composition and neurotransmitter concentration in brain of neonatal rat. The correlation of the level of AA and the content of GLN (**a**); The correlation of the level of AA and the content of 5-HIAA (**b**); The correlation of the level of AA and the ratio of 5-HIAA/5-HT (**c**); The correlation of the level of DHA and the content of GLN (**d**); The correlation of the level of DHA and the content of 5-HIAA (**e**); The correlation of the level of DHA and the ratio of 5-HIAA/5-HT (**f**); The correlation of the ratio of DHA/AA and the content of GLN (**g**); The correlation of the ratio of DHA/AA and the content of 5-HIAA (**h**); The correlation of the ratio of DHA/AA and the ratio of 5-HIAA/5-HT (**i**)

A significant interaction of brain AA, DHA status, DHA/AA ratio and neurotransmitter concentrations was detected. Pearson correlation analysis revealed that the brain level of AA was negatively correlated with the content of GLN (Fig. 3a, *r* = 0. 4265, *P* < 0.01), 5-HIAA (Fig. 3b, *r* = 0. 6061, *P* < 0.01) and the ratio of 5-HIAA/5-HT (Fig. 3c, *r* = 0. 6128, *P* < 0.01). The brain level of DHA was positively correlated with the content of GLN (Fig. 3d, *r* = 0. 7352, *P* < 0.01), 5-HIAA (Fig. 3e, *r* = 5950, *P* < 0.01) and the ratio of 5-HIAA/5-HT (Fig. 3f, *r* = 0. 6751, *P* < 0.01). Similarly, the ratio of DHA/AA was also positively correlated with the content of GLN (Fig. 3g, *r* = 0.7732, *P* < 0.01), 5-HIAA (Fig. 3h, *r* = 0.7200 *P* < 0.01) and the ratio of 5-HIAA/5-HT (Fig. 3i, *r* = 0. 8297, *P* < 0.01).

## Discussion and conclusions

Fatty acid (FA) composition affects the physicochemical properties of the membrane, thus influences the conformation and function of membrane-bound proteins, such as receptors, ion channels, and transporters [11]. LC-PUFA also serve as precursors for inter- and intracellular signals such as prostaglandins and thromboxanes, and modulate the gene expression by activating of transcription factors. Moreover, accumulating studies have shown that maternal diets deficient in n-3 PUFAs decreased the brain DHA content of offspring [12], while the deficiency in brain DHA may represent a preventable neurodevelopmental risk factor for the subsequent emergence of psychopathology. In the present study, we discovered that diverse maternal n-3 PUFAs dietary altered the FA composition of offsprings in liver and brain. Maternal deficient n-3 diet decreased the percentages of DHA, total n-3 PUFAs, and increased the percentage of total n-6 PUFAs in the liver and brain of neonatal female rats compared with control group. Since brain DHA is pivotal for the neurodevelopment, and deficiency on DHA may aggravate symptoms of anxiety and depression, adequate of N-3 PUFAs during pregnancy is essential for the development of rats.

Neurogenesis is established during gestation in most brain region and is completed before birth except in the dentate gyrus (DG) of hippocampal [13]. The normal development of DG is very important to learning and memory [14]. N-3 PUFAs was reported to have neurogenesis-promoting properties [15]. Contrarily, maternal dietary n-3 fatty acid deficiency can impair normal neurogenesis in the embryonic rat brain [16]. In the present study, we first found that maternal n-3 PUFAs deficiency significantly decreased the number of BrdU^+^ cells in the hippocampus DG in neonatal rats. This inhibition of proliferation might reduce the neural progenitor pool and affect subsequent neurogenesis, further leading to the impaired learning and memory and declined in cognitive abilities.

It has been postulated that the alterations of neurotransmission in the key brain areas play a pivotal role in the progression of several neuropsychiatric diseases, and the beneficial effects of n-3 PUFAs in these brain-related disorders was, at least partially, via its modulating effect on neurotransmission [17]. GABA is the major inhibitory neurotransmitter in the central nervous system. GABAergic neurons have been shown to control cognitive processing in prefrontal and hippocampal networks [18]. Recent study suggested that reduced brain DHA could decrease presynaptic GLU release and uptake, further resulted in degradation of glutamatergic transmission in hippocampus of aging rats, rather than young rats [6]. GABA is produced in the CNS via decarboxylation of GLU, in a reaction catalyzed by glutamic acid decarboxylase (GAD). Within the CNS, the majority of GLU is produced from glutamine (GLN) via the enzyme glutaminase. In the present study, maternal n-3 PUFAs deficiency significantly decreased the brain concentration of GLN and increased the content of GABA in offspring rats, without altering the level of GLU and the mRNA expression of GAD. Maternal n-3 PUFAs Deficiency gave rise to the failure of the GGC cycle (inhibition of GLN synthesis) in neonatal female rats. We speculated that the decreased GLN in Deficient group might reflect the degradation of glutamatergic transmission and GABAergic transmission, and the increase in GABA content may be the compensatory mechanism in the organism.

The altered tissue FA composition induced by dietary n-3 PUFAs content could affect CNS dopamine systems [19]. Current studies between the relationship of n-3 PUFAs level and FA composition focused on the dopamine receptors, which proposed that inadequate n-3 PUFAs diet could increase densities of D_1_ and D_2_ receptors, but had no effect on DA content [20]. In line with previous findings, the content of DA in the neonatal female rats of n-3 PUFAs deficient group remained stable, as well as the expression of tyrosine hydroxylase (TH), the key synthase enzymes of DA. However, the concentrations of DOPAC and HVA, the primary metabolites of DA, were concurrently elevated. It is worth noting that maternal n-3 PUFAs supplementary significantly reduced the brain DA level and significantly increased the DOPAC content in neonatal female rats. Since DA plays a key role in governing motivation and reward processing, and alterations to dopamine levels have been linked to the onset of various psychiatric disorders including schizophrenia, substance abuse disorders, attention-deficit/hyperactivity disorder, and depression [21], adequate maternal n-3 PUFAs diet become essential for normal brain development.

The role of n-3 PUFAs in modulation of noradrenergic neurotransmission has received relatively little attention. In animal studies, a diet containing inadequate n-3 PUFAs resulted in a decreased norepinephrine (NE) in the hippocampus, cortex and striatum of rats [22]. However, no alterations in regional NE reduction were found in virgin female or postpartum female rats with brain DHA deficiency [23, 24]. Similarly, the brain NE level of neonatal female rats remained stable in maternal n-3 PUFAs deficiency group, but its metabolite VMA decreased significantly. Unexpectedly, we first found that maternal n-3 PUFAs supplementary could reduce the content of NE without altering the metabolites like VMA and MHPG in neonatal female rats. We assume that the reduced NE content in the brain may result from the reduction of its precursor DA.

N-3 PUFAs deficiency during peri-adolescent (P21-90) was reported to exhibit significantly greater 5-hydroxytryptamine (5-HT) content and a significantly smaller 5-HIAA/5-HT ratio in the prefrontal cortex and hypothalamus of female adult rats [4, 9]. Furthermore, the effect of DHA efficiency on 5-HT turnover was depended on when dietary n-3 fatty acid deficiency was initiated, and there might be a neurodevelopmental window in which 5-HT system was vulnerable to dysregulation in response to DHA deficiency. Similar to previous results, we found that maternal n-3 PUFAs deficiency not only slightly increased 5-HT content and significantly reduced 5-HIAA/5-HT ratio, but also significantly reduce 5-HIAA content in neonatal female rats. On the contrary, maternal n-3 PUFAs supplementary significantly increased the concentration of 5-HIAA and the ratio of 5-HIAA/5-HT. The rate-limiting enzyme in the biosynthesis of 5-HT in the brain is tryptophan hydroxylase 2 (TPH2). In addition to TPH2, tryptophane (TRY), the precursor of 5-HT, also can be metabolized by indoleamine 2,3-dioxygenase (IDO) which is activated by inflammatory cytokines resulting in accelerated conversion of TRP to kynurenine (KYN) and reduced bioavailability of TRP for 5-HT production [25], whereas n-3 PUFAs was reported to participate in various neuro-immune regulation [26, 27]. Thus, it is possible that n-3 PUFAs levels may affect IDO activity via its immunomodulating effects. In the present study, n-3 PUFAs supplementary slightly increased TRY and significantly increased KYN content. Whereas, the brain levels of TRY and KYN were unchanged in n-3 PUFAs deficiency diet, indicating that maternal n-3 PUFAs levels appeared to exert no effect on KYN pathway in the brain of neonatal rats.

Accumulating evidence suggests that neurotransmitter system play a vital role in neurogenesis. Antidepressant treatment with SSRIs, which would increase the content of 5-HT in synaptic cleft, was reported to promote cell proliferation and neurogenesis in models of depression [28]. Furthermore, in vivo study suggested that serotonin plays a direct and acute regulatory role in activity-dependent hippocampal neurogenesis [29]. Besides, the reduction of GABA-ergic interneurons is thought to be responsible for the aberrant neurogenesis [30]. In the present study, both serotonin system and glutamatergic system of neonatal female rats were altered, and highly associated with the content of AA, DHA and the ratio of DHA/AA. Based on above studies, it is easily to come to the conclusion that the inadequate intake of N-3 PUFAs could disturb the GABA system and 5-HT metabolites, further impairing hippocampal neurogenesis.

In conclusion, our study showed that maternal deficiency of n-3 PUFAs might have considerable effects on central nervous system of neonatal female rats mainly through impairing the normal neurogenesis, glutamatergic system and 5-HT turnover, which suggest that adequate n-3 PUFA during pregenancy is essential for fetal neurodevelopment. However, more research is needed to evaluate the long-term effect.

## Methods

### Animals and husbandry

This study was approved by the Animal Care & Use Committee of Central South University. All experiments were performed in accordance with the Guide for Care and Use of Laboratory Animals (Chinese Council).

Sprague-Dawley rats were initially housed in groups in a temperature-controlled environment under a 12/12 h light/dark cycle with *ad libitum* access to food and water. Rats were randomly assigned to three groups (*n* = 7) according to the content of n-3 PUFAs in their diets: Deficient, Control and Supplementary. Breeding stock (Female, 210-230 g; Male proven breeders; The Experimental Animal Center of the Second Xiangya Hospital) maintained on corresponding diets two weeks before mating (Female 280-300 g) until the end of the experiment. At the time of mating, one male rat was housed with two female rats per cage for three days. To meet all current nutrient standards for rat pregnancy and growth [31], the Control diet in our experiment was AIN-93G (Trophic Animal Feed High-Tech Co., Ltd, China) formulated with soybean oil (70 g/kg). The Deficient and Supplementary diet were identical to the Control diet except the oil formulation. The Deficient diet was prepared with safflower oil (70 g/kg) and the Supplementary diet was prepared with fish oil (20 g/kg) and soybean oil (50 g/kg). Fatty acid composition of the diets is shown in Table 4. The nutritional composition of diets and the fatty acid composition of fish oil are shown in Additional file 1: Tables S1 and S2. Chromatogram of fish oil is also provided in Additional file 2: Figure S1.

**Table 4:** Fatty acid composition of the Experimental Diets

Fatty acid	Content in diet(area percent)		
	Deficient	Control	Supplementary
C16:0	6.61	11.18	9.96
C18:0	2.31	3.17	3.39
C18:3n3	ND	4.26	3.67
C18:1n9c	11.71	24.52	22.72
C18:2n6c	79.38	55.39	47.30
C20:5n3	ND	ND	7.32
C22:6n3	ND	ND	4.00
Other MUFA	ND	1.48	1.95

Diet fatty acid composition was determined by GC/MS using Supelco 37 Standard. ND: Not detected

### Bromodeoxyuridine treatment

To determine whether maternal n-3 FUFAs status affects the cell proliferation in neonatal female rats, bromodeoxyuridine ((+)-5’-bromo-2’-deoxyuridine [BrdU]; Sigma-Aldrich, USA) in 0.9 % sterile saline solution was injected intraperitoneally at dose of 100 mg/kg. After 2 hour, rats were sacrificed and brains were removed and fixed overnight in 4 % paraformaldehyde. After then, tissues were cryoprotecter in 30 % sucrose in PBS before embedding. Immunefluorescence of BrdU-labeled nuclei was measured using BrdU Assay kit (servicebio, China) according to the protocol.

### FA composition assays

A classical method for lipid extraction and purification was applied with modification [32]. Briefly, 750 μl mixture of dichloromethane and methanol (2:1) and 100 μM butylated hydroxytoluene (to prevent lipid peroxidation) were added to the brain tissues, and homogenized by tissue homogenizer. The mixture was then added with 250 μl dichloromethane and followed with blending for 30 s, and 250 μl water was added and blending was continued for another 30 seconds. The lower phase was then transferred and subsequently evaporated to dryness by nitrogen. The residue was dissolved by n-hexane, and then 2 ml of 0.5 M KOH-MeOH was added, the sample was heated at 60 °C for 20 min. Following 10 min of cooling period, 3 ml of 12.5 % H_2_SO_4_ in methanol was added to methylate the sample. After an additional 60 min of heating in the water bath (60 °C), the sample vial was allowed to cool, and 1 ml of saturated solution containing sodium chloride and 2 ml of hexane was added. The hexane fraction was then transferred for GC analysis. Total fatty acid composition was determined with Agilent 7890A/5975C. The column was VF-23 ms (Agilent): 30 m, (length), I.D. 0.25 mm wide bore, film thickness of 0.25 μM. Fatty acid identification was determined using retention times of authenticated fatty acid methyl ester standards (Supelco 37, sigma). Fatty acid composition data was expressed as percentage of peak area.

### The determination of neurotransmitters

Neurotransmitters and their metabolites were quantified using high-performance liquid chromatography coupled to tandem mass spectrometry (HPLC-MS/MS) as previously described [33] with little modification. Briefly, brain tissues were homogenized by tissue homogenizer with 1 ml of 85 % ice-cold acetonitrile-water adding 10 μl of mixed internal standard solution (containing 20 μg/ml 3,4-dihydroxybenzylamine, 10 μg/ml 5-hydroxyindole-2-carboxylic acid and 100 μg/ml L-aspartic acid-13C4,15 N). After the homogenate, the mixture was centrifuged at 4 °C for 15 min at 10000 rpm. The supernatant (500 μL) was then transferred and subsequently evaporated to dryness. For derivatization, 150 μl of dansyl chloride solution (4 mg/ml in acetonitrile) and 50 μl of 0.1 M Na_2_CO_3_-NaHCO_3_ buffer (pH 11.0) were added to the residue and reacted at 35 °C for 30 min. After the reaction, the pH of the mixture was adjusted by adding 10 μl of 7.5 % formic acid solution. After centrifugation, the supernatant was transferred to the vial for analysis. HPLC-MS/MS analysis was carried out on a Waters Acquity ultra-performance liquid chromatography system (Waters, USA) with a Micromass Quattro Premier XE tandem quadruple mass spectrometer (Waters, USA) equipped with ESI source. The chromatographic separation was achieved on Ultimate XB-C8 column, 2.1 mm × 50 mm, 3.0 μm particle size (Welch, China). The mobile phase for elution was a gradient established between solvent A (water with 20 mM ammonium acetate and 0.1 % formic acid) and solvent B (acetonitrile) at a flow rate of 0.25 ml/min. The mass spectrometer was operating at the following parameters: capillary voltage, 3.00 kV; extractor voltage, 3.00 V; source temperature, 120 °C; desolvation temperature, 450 °C; desolvation gas flow, 750 L/h; cone gas flow, 50 L/h. Argon used as the collision gas was introduced into the collision cell at a flow rate of 0.16 ml/min. The electrospray ionization source was operated in the positive mode. Data acquisition was carried out by Mass Lynx 4.1 software. Neurotransmitters were quantified relative to the internal standard areas and calibrated using standard curves.

### Real-time PCR analysis

Total RNA from the brain was homogenized using Trizol reagent (invitrogen, USA) according to the manufacturer’s instructions. The real-time reverse transcriptase polymerase chain reaction (RT-PCR) was used to examine the effect of maternal n-3 PUFAs deficiency or supplementary on certain key synthase enzymes of neurotransmitters in neonatal rats. Quantification of mRNAs was performed on ABI 7900HT Detection System (Lifetech, USA) using SYBR® Premix Ex TaqTM (Takara RR820A, China) and gene-specific primers. Relative quantitation for PCR product was normalized to β-actin mRNA values obtained from the same tissue. The sequences of gene- specific primers are provided in Table 5.

**Table 5:** Primer sequences used for the real-time PCR analysis

Gene (accession no.)	Sense Primer (5’-3’)	Antisense Primer (5’-3’)	Amplicon length
GAD65 (NM_012563.1)	CAGCCTGTGAAGGAGAAAGG	GGTCTGCCAATTCCCAATTA	159 bp
GAD67 (M76177.1)	CTGGAGCTGGCTGAATACCT	TCGGAGGCTTTGTGGTATGT	146 bp
TPH2 (NM_173839.2)	GGGTTACTTTCCTCCATCGGA	AAGCAGGTTGTCTTCGGGTC	119 bp
GS (M29579.1)	CCACTGTCCCTGGGCTTAGTTTA	AGTGACATGCTAGTCCCACCAA	85 bp
TH (NM_012740.3)	CTCCTCACCTATGCATTCAC	TCCAATGTCCTGGGAGAACT	105 bp
β-Actin (NM031144)	CATCCTGCGTCTGGACCTGG	TAATGTCACGCACGATTTCC	116 bp

### Statistical analysis

Results from the experiment were expressed as means ± SEM and analyzed using SPSS software. Differences between groups were determined by one-way analysis of variance (ANOVA) for multiple comparisons followed by TUKEY or Dunnett’s t test for post-hoc comparisons. Pearson correlation analysis was used to evaluate possible associations between certain FA and neurotransmitters. A *p* < 0.05 was considered statistically significant.

## Additional files

Additional file 1: Table S1. Nutritional Composition of Diets. Table S2 Fatty acid Composition of fish oil. (DOCX 13 kb) Additional file 2: Figure S1. Chromatogram of fish oil. (JPG 387 kb)

## Abbreviations

5-HIAA: 5-hydroxy indole acetic acid
5-HT: serotonin
AA: arachidonic acid
ANOVA: analysis of variance
BrdU: bromodeoxyuridine
DA: dopamine
DG: dentate gyrus
DHA: docosahexaenoic acid
DOPAC: 3,4- dihydroxyphenyl acetic acid
EPA: eicosapentaenoic acid
FA: fatty acid
GABA: γ-aminobutyric acid
GAD: glutamate decarboxylase
GLN: glutamine
GLU: glutamate
GS: glutamine synthetase
HVA: homovanillic acid
KYN: kynurenic acid
MHPG: 4-Hydroxy-3-methoxyphenylglycol
NE: norepinephrine
PUFAs: Long-chain polyunsaturated fatty acids
TH: tyrosine hydroxylase
TPH: tryptophan hydroxylase
TRP: tryptophan
VMA: vanilmandelic acid
