# Supplementary material for: Maternal diet of polyunsaturated fatty acid altered the cell proliferation in the dentate gyrus of hippocampus and influenced glutamatergic and serotoninergic systems of neonatal female rats
Source: Lipids Health Dis. 2016 Apr 5;15:71. doi: 10.1186/s12944-016-0236-1 (PMC4822267; doi:10.1186/s12944-016-0236-1)
Supplement: Additional file 1: Table S1. — Nutritional Composition of Diets. Table S2 Fatty acid Composition of fish oil. (DOCX 13 kb) [file 12944_2016_236_MOESM1_ESM.docx]

**Additional file 1**

**Table S1***.* Nutritional Composition of Diets

| Ingredient | Low (gm） | Control (gm） | High(gm） | |
| --- | --- | --- | --- | --- |
| Casein，30 Mesh | 200 | 200 | 200 |  |
| L-Cystine | 3 | 3 | 3 |  |
| Corn Starch | 397 | 397 | 397 |  |
| Maltodextrin | 132 | 132 | 132 |  |
| Sucrose | 100 | 100 | 100 |  |
| Cellulose | 50 | 50 | 50 |  |
| Mineral Mix | 35 | 35 | 35 |  |
| Vitamin Mix | 10 | 10 | 10 |  |
| Choline Bitartrate | 2.5 | 2.5 | 2.5 |  |
| t-Butylhydroquinone | 0.014 | 0.014 | 0.014 |  |
| soybean oil | 0 | 70 | 50 |  |
| Safflower oil | 70 | 0 | 0 |  |
| Fish oil | 0 | 0 | 20 |  |

**Table S2.** Fatty acid Composition of fish oil

| Fatty acid | Content in fish oil |
| --- | --- |
| C16:0 | 2.734% |
| C18:0 | 2.699% |
| C18:1n9t | 11.110% |
| C18:1n9c | 2.719% |
| C18:2n6c | 12.091% |
| C20:5n3 | 34.344% |
| C22:6n3 | 25.011% |
