# Supplementary figures and images for: Maternal diet of polyunsaturated fatty acid altered the cell proliferation in the dentate gyrus of hippocampus and influenced glutamatergic and serotoninergic systems of neonatal female rats
Source: Lipids Health Dis. 2016 Apr 5;15:71. doi: 10.1186/s12944-016-0236-1 (PMC4822267; doi:10.1186/s12944-016-0236-1)

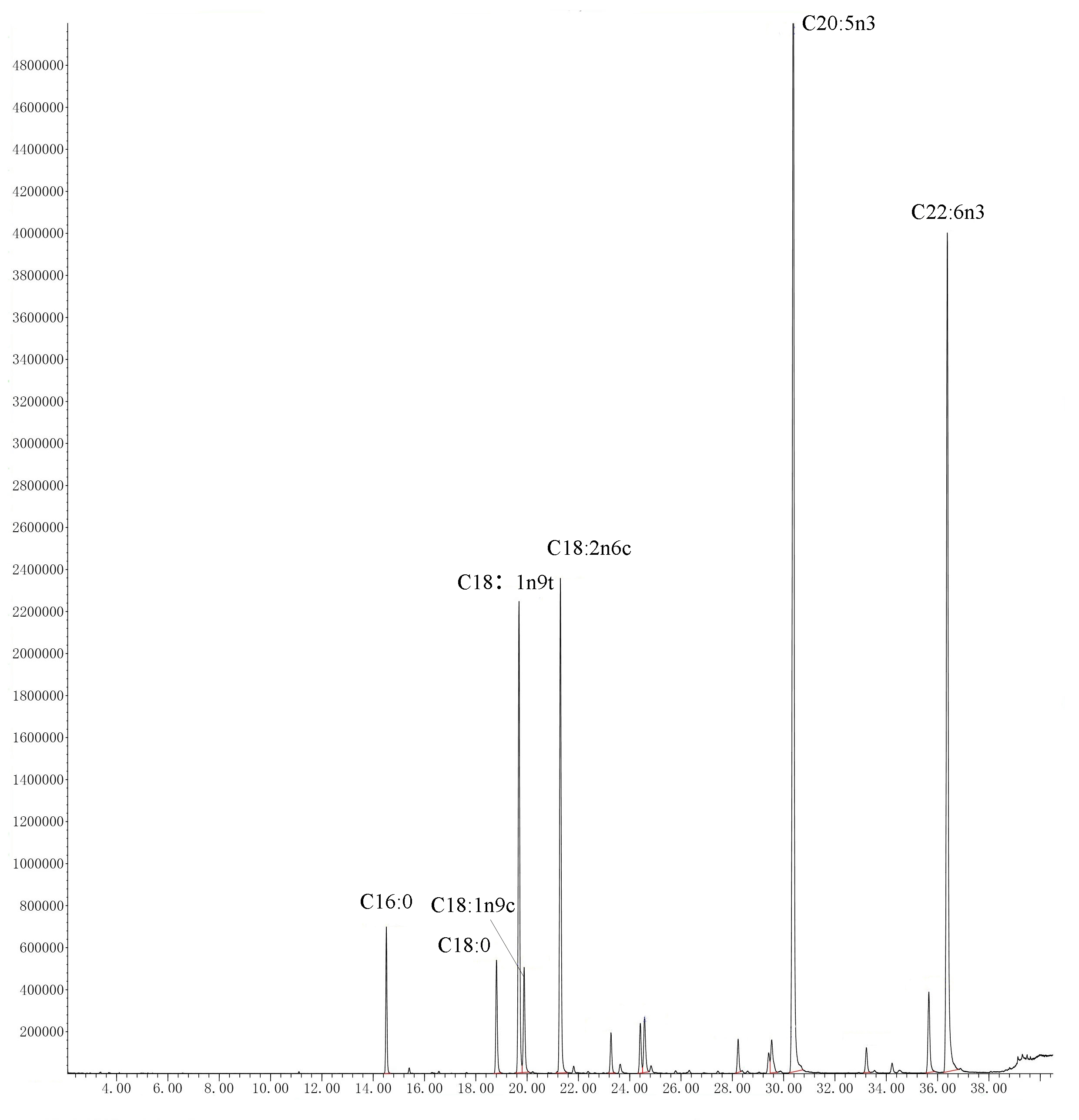

Supplement: Additional file 2: Figure S1. — Chromatogram of fish oil. (JPG 387 kb) [file 12944_2016_236_MOESM2_ESM.jpg]
